# Silencing of *SlMYB78-like* Reduces the Tolerance to Drought and Salt Stress via the ABA Pathway in Tomato

**DOI:** 10.3390/ijms252111449

**Published:** 2024-10-24

**Authors:** Yu Liu, Pengyu Guo, Zihan Gao, Ting Long, Chuanji Xing, Jing Li, Jing Xue, Guoping Chen, Qiaoli Xie, Zongli Hu

**Affiliations:** 1 Laboratory of Molecular Biology of Tomato, Bioengineering College, Chongqing University, Room 521, Campus B, 174 Shapingba Main Street, Chongqing 400044, China; 18436099626@163.com (Y.L.); gzhfilm@163.com (Z.G.); longting325112@163.com (T.L.); micy180605@163.com (J.L.); chenguoping@cqu.edu.cn (G.C.); qiaolixie@cqu.edu.cn (Q.X.); 2 Institute of Grassland, Flowers and Ecology, Beijing Academy of Agriculture and Forestry Sciences, 11 Shuguanghuayuan Middle Road, Haidian, Beijing 100097, China; 18704404680@163.com (C.X.); xuejing@baafs.net.cn (J.X.)

**Keywords:** tomato, *SlMYB78-like*, drought and salt stresses, ABA

## Abstract

The MYB transcription factor family plays a crucial regulatory role in plant growth, development, biological progress, and stress responses. Here, we identified a R2R3-MYB transcription factor gene, *SlMYB78-like*, from tomato and characterized its function by gene silencing via RNA interference (RNAi). The results exhibited that the silencing of *SlMYB78-like* reduced the sensitivity of tomato seedlings to exogenous ABA. In addition, when exposed to drought and salt stresses, the RNAi lines grown in soil showed decreased tolerance, with lower ABA accumulation, relative water content, and chlorophyll content while displaying higher relative conductivity and malondialdehyde (MDA) content than the wild type. Moreover, the expression of genes related to chlorophyll biosynthesis, photosynthesis, and ABA biosynthesis/response were down-regulated in *SlMYB78-like*-silenced lines. Notably, the transcript level of *SlCYP707-A2*, which encodes a protein involved in ABA degradation, was up-regulated significantly after stresses. The transient expression assay Dual-luciferase (Dual-LUC) and a yeast one-hybrid (Y1H) assay demonstrated that SlMYB78-like bound to the promoter of *SlCYP707-A2*. Additionally, the physical interaction between SlMYB78-like and SlDREB3, which functioned in ABA signaling transduction, was identified through yeast two-hybrid (Y2H) and bimolecular fluorescence complementation (BiFC) assays. Collectively, our study illustrates that *SlMYB78-like* participates in the abiotic stress response via the ABA pathway.

## 1. Introduction

MYB transcription factors constitute one of the largest transcription factor families in plants, exhibiting widespread distribution across various plant species. They possess a highly conserved N-terminal MYB DNA-binding domain with repeat sequences (Rs) [1], while the C-terminal region displays significant variability, contributing to the functional diversity of the MYB family genes. Based on the position and number of these repeat sequences, MYB transcription factors are categorized into four types: 1R (R1/2, R3-MYB), 2R (R2R3-MYB), 3R (R1R2R3-MYB), and 4R [2]. Among these, the 1R MYB transcription factors primarily maintain chromosomal structure integrity and regulate the transcription levels of associated genes. The 2R MYB transcription factors represent the most numerous and functionally complex group within the MYB family, playing crucial roles in cell differentiation, response to abiotic stress, and regulation of secondary metabolite production. Notably, numerous 2R MYB transcription factors exhibit transcriptional self-activation activity, as documented in the literature. The 3R MYB transcription factors influence cell division and differentiation processes through cell cycle regulation [3,4]. The 4R MYB transcription factors are the least common and their functions remain relatively less explored [5].

Numerous studies have demonstrated the diverse functions of MYB transcription factors in plants, including roles in plant growth regulation, hormone signal transduction, secondary metabolism, as well as responses to biotic and abiotic stresses, and so on [5,6,7,8]. For instance, *AtMYB17* and *LFY* are involved in early inflorescence development and seed germination [9], while *AtMYB59* influences the growth and development of *Arabidopsis* by controlling the cell cycle in root apical cells and inhibiting root elongation [10]. AtMYB2 governs the expression of drought response genes which are induced by abscisic acid (ABA) [11]. AtMYB49 enhances salt tolerance by increasing the thickness of the cuticle through the activation of cuticle and wax-related genes [12]. A study found that the apple MYB4, involved in flavanol synthesis, targets lignin synthesis genes such as *CAD*, promoting the synthesis of lignin and flavanols [13]. *MtMYB2* acts as a pigment repressor gene in the leaves and seed coats of *Medicago truncatula*, negatively regulating anthocyanin or proanthocyanidin synthesis by forming an MBW complex [14]. *Arabidopsis* AtMYB118 induces the expression level of genes related to seed endosperm formation, thereby regulating the content of embryo storage proteins and affecting seed viability and germination [15]. Research has identified that MYB109 in cotton regulated the expression of downstream genes, such as *GhTUB1* and *GhACO2*, to promote the formation of longer fibers in transgenic plants, further increasing crop yields [16]. These findings highlight the significant roles played by MYB transcription factors in various aspects of plant growth and development.

Drought and salt stress are common abiotic factors which restricted plant growth, development, and crop yield in natural environments. In response to these stresses, plants alter endogenous plant hormone contents, accumulate of metabolites, and modify gene transcript levels, with transcription factor playing a crucial role. Accumulating evidence has characterized the significant involvement of transcription factor involved in stress responses. For example, the silencing of *JUB1*, a NAC transcription factors gene in tomato, inhibits tolerance to drought. JUB1 targets and combines with the promoter of *SlDREB1*, *SlDREB2*, and *SlDELLA* to alter the plant’s stress response [17]. Similarly, the knockout of *OsbHLH38* in rice increases sensitivity to salt stress, as OsbHLH38 regulates the transcription level of genes involved in photosynthesis, redox homeostasis, and abiotic stress responsiveness [18]. In tobacco, NtERF13a positively promotes the tolerance to salt/drought stress, and its binding with the promoter of *NtHCT*, *NtF3′H*, *NtANS* has been identified [19]. Conversely, as a negative regulator in salt and drought stress response, the overexpression of *GmbZIP19* of soybean in *Arabidopsis* reduces tolerance to abiotic stresses and influences the transcript level of ABA maker genes [20]. Furthermore, *Arabidopsis* lines overexpressing *HbWRKY82* (from *Hevea brasiliensis*) lines exhibit improved the resistance to dehydration and salinity, together with the decreased sensitivity to exogenous ABA [21].

Recent research has characterized MYB proteins as crucial transcription factors involved in abiotic stress response. For instance, the overexpression of *SbMYBAS1*, a negative regulator of abiotic stress response, in *Arabidopsis* results in decreased tolerance to salt stress by altering the expression level of genes including *AtGSTU17*, *AtGSTU16*, *AtP5CS2*, *AtUGT88A1*, *AtUGT85A2*, *AtOPR2*, and *AtPCR2* [22]. Under salt stress conditions, the overexpression of *MdMYB108L* in *Arabidopsis* exhibits an increased resistance to abiotic stress, and MdMYB108L was found to bind to the promoter of *MdNHX1*, a salt tolerance gene [23]. In tobacco, the overexpression of *IbMYB308* from sweet potato, which is induced by several abiotic stresses, enhanced salt stress tolerance, illustrating its positive role in stress response [24]. *Arabidopsis* lines overexpressing-*HbMYB44* from Rubber tree exhibited improved tolerance to abiotic stress, and it recovered from root growth inhibition caused by exogenous phytohormones [25]. Similarly, overexpressing *CgMYB1* (*Chenopodium glaucum*) in *Arabidopsis* enhances tolerance to salt stress. Moreover, the interaction between CgMYB1 and CgbHLH001 was shown to activate the expression of downstream stress-responsive genes [26].

Tomato (*Solanum lycopersicum*), with its exceptional nutritional and commercial value, is considered one of the most important vegetable crops and serves as a representative model plant. So far, there are 127 MYB members in the tomato genome, comprising 122 R2R3-, 4 R1R2R3-, and 1 4R-MYB proteins [27]. Several of these MYB members have been identified as important regulators in stress response. For example, the silencing of *SlMYB55* mediated by RNA interference (RNAi) has been shown to enhance tolerance of transgenic lines to drought/salt stress by modifying the ABA biosynthesis and signal transduction [28]. Similarly, the *SlMYB50*-RNAi lines also exhibited enhanced tolerance to salt/drought stress and the interaction of SlMYB50 with the promoter of *CHS1* influenced the biosynthesis of flavonoid, thereby further regulating abiotic stress tolerance [29]. The overexpressed *SlMYB49* tomato lines display increased tolerance to drought and salt stresses, illustrating its positive function involved in stress response [30]. Moreover, the overexpression of *SlMYB102* confers tolerance to low-temperature and salt stress [31,32].

In the present study, we characterized the function of *SlMYB78-like* (*Solyc05g053330*), which belongs to the R2R3-MYB subfamily, via RNAi. The results demonstrate that the expression of *SlMYB78-like* was induced by drought, salt stress, and exogenous ABA. When exposed to salt and drought stress conditions, the *SlMYB78-like*-RNAi tomato lines exhibit reduced tolerance compared to wild-type plants, accompanied by a lower accumulation of endogenous ABA. Furthermore, Dual-Luciferase reporter assays and yeast one-hybrid experiments revealed that the SlMYB78-like protein targeted and regulated the transcriptional activity of its downstream target gene, *SlCYP707-A2*. Yeast two-hybrid and bimolecular fluorescence complementation assays characterized the physical interaction between SlMYB78-like and SlDREB3. This study elucidated the role of the tomato *SlMYB78-like* gene in drought and salt stress response, providing a theoretical foundation for the development of tomato stress-resistant breeding programs.

## 2. Results

### 2.1. Molecular Characterization of SlMYB78-like

**Figure 1:**
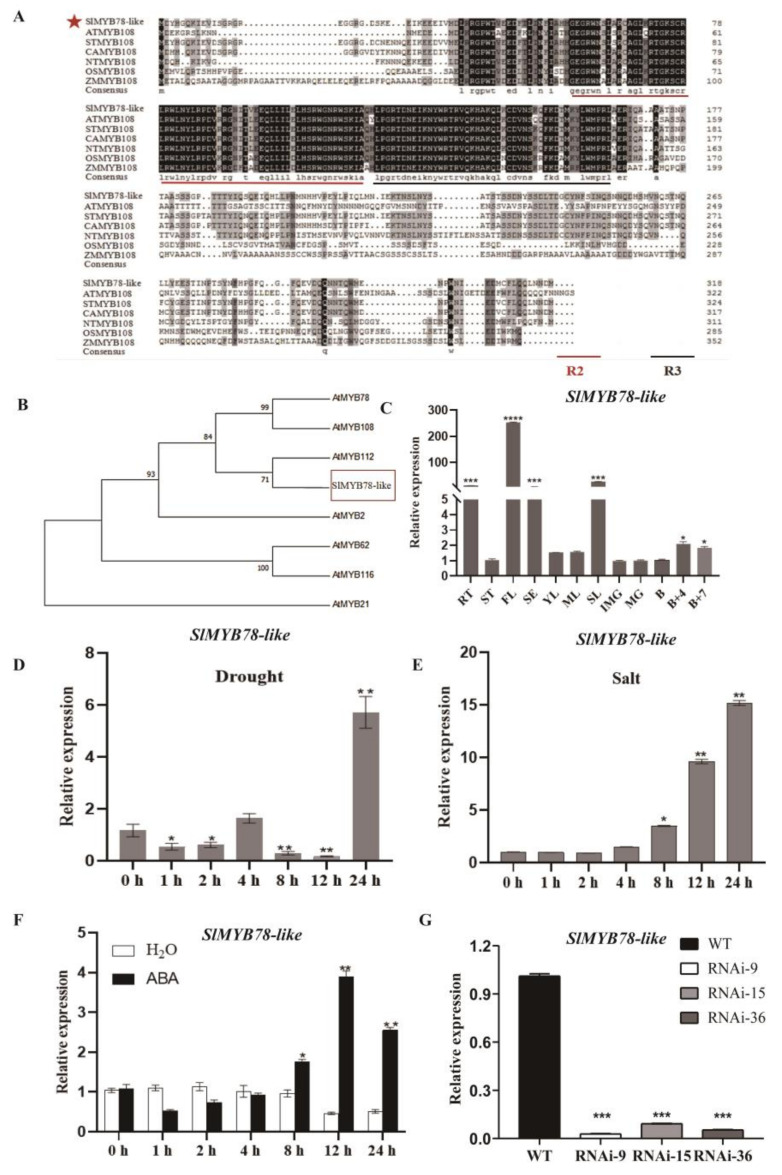
(**A**) Multiple sequence alignment of SlMYB78-like and homologous proteins in other species. Red star is used to indicate the target genes in this study, *SlMYB78-like*. (**B**) Phylogenetic analysis of SlMYB78-like and some MYB proteins in *Arabidopsis* was constructed by the neighbor-joining method and a bootstrap analysis of 1000 replicates. The accession numbers for the proteins are listed as follows: SlMYB78-like (XP_004239882); AtMYB78(NP_001190502); AtMYB108(NP_187301); AtMYB112(NP_564519); AtMYB2(NP_182241); AtMYB62(NP_176999); AtMYB116(NP_001031091); AtMYB21(NP_189418). (**C**) The expression of *SlMYB78-like* in RT, roots; ST, stems; YL, young leaves; ML, mature leaves; SL, senescent leaves; FL, flowers at anthesis; SE, sepal; IMG, immature green fruits; MG, mature green fruits; B, breaker fruits; B + 4, 4 days after breaker fruits; B + 7, 7 days after breaker fruits in WT. (**D**,**E**) Expression patterns of *SlMYB78-like* under the dehydration and salinity treatments. (**F**) Expression patterns of *SlMYB78-like* in response to ABA treatments. The 30-day-old tomato seedlings were sprayed with water and 100 μM ABA solution, respectively, and the leaves were collected at the indicated times. (**G**) The qRT-PCR results exhibited the inhibited expression level of *SlMYB78-like*-RNAi lines compared with the wild type for further research. All data were means (±SE) of three independent biological replicates (* *p* < 0.05, ** *p* < 0.01, *** *p* < 0.001, **** *p* < 0.0001).

Gene sequence analysis showed that *SlMYB78-like* (Solyc05g053330) processed an open reading frame (ORF) of 957 bp, encoding a protein of 318 amino acid residues (Table S3). Multiple protein sequence alignments revealed that the SlMYB78-like protein contained R2, R3 repeats in the N-terminal region, classifying it within the R2R3-type MYB subfamily (Figure 1A). An analysis of the *SlMYB78-like* promoter identified various *cis*-regulatory elements, including the CAACTG-motif responsive to dehydration, the ACGTG-motif responsive to ABA, and the TACGTG-motif responsive to light, indicating that *SlMYB78-like* might be intimately associated with abiotic stress response (Table S2). A phylogenetic tree, constructed using MEGA (Molecular Evolutionary Genetics Analysis, version 6.06), indicated that SlMYB78-like exhibited the closest evolutionary relationship to AtMYB112, followed by AtMYB78 and AtMYB108 (Figure 1B).

### 2.2. Expression Pattern Analysis of SlMYB78-like and Obtaining of SlMYB78-like-RNAi Lines

Employing qRT–PCR, we found that the expression level of *SlMYB78-like* in wild-type (WT) plants was highest in flowers, followed by senescent leaves, sepals, and roots, with lower expression in other parts (Figure 1C). Following drought treatments, the expression levels of *SlMYB78-like* in WT tomato increased to the highest point at 24 h, which was approximately 7-fold higher than that at 0 h (Figure 1D). Moreover, the transcription levels of *SlMYB78-like* showed a general increasing trend after salt stress (Figure 1E). These results indicated that *SlMYB78-like* may be involved in the response to salt and drought stress. In addition, after ABA treatment, the mRNA accumulation of *SlMYB78-like* showed an upward trend, reaching its peak point at 12 h (Figure 1F), indicating that *SlMYB78-like* responds to ABA.

To further investigate the function of *SlMYB78-like*, an RNAi vector targeting *SlMYB78-like* was constructed. Subsequently, three independent silenced lines (RNAi09, RNAi15, and RANi36 lines) exhibiting decreased expression levels of *SlMYB78-like* were generated for further investigation (Figure 1G).

### 2.3. Silencing of SlMYB78-like Affected ABA Response

**Figure 2:**
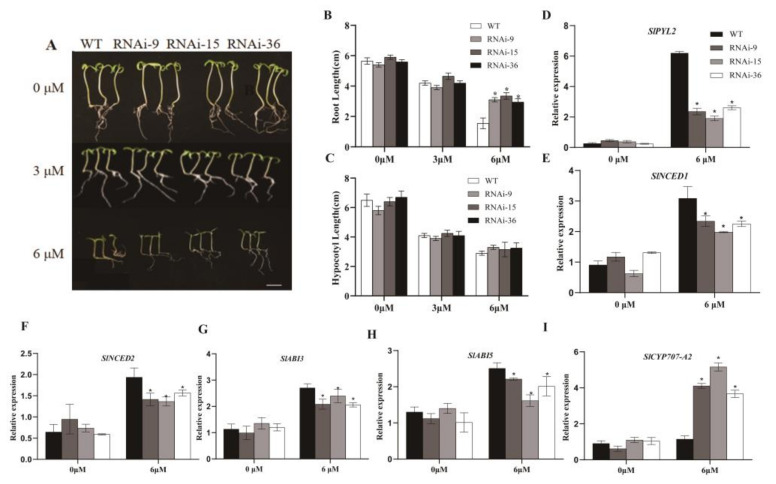
(**A**) Phenotypes of WT and SlMYB78-like-RNAi seedlings under different-concentration ABA treatments. (**B**,**C**) Comparison of root length and hypocotyl length of WT and *SlMYB78-like*-RNAi seedlings after ABA treatment. (**D**–**I**) Transcript levels of *SlCYP707-A2* (ABA degradation gene), *SlPYL2* (ABA receptor gene), *SlABI3*/*5* (ABA response genes), and *SlNCED1*/*2* (ABA biosynthesis genes) in wild-type and transgenic lines before and after ABA treatment. The scale bar represented 1 cm. WT: wild type; ±SE: standard error. All data are presented as means (±SE) from three independent biological replicates. Asterisks above each column indicate significant differences between WT and transgenic lines (* *p* < 0.05).

The bioinformatics analysis revealed that the promoter of *SlMYB78-like* contained ABA-responsive elements (Table S2), and the expression pattern showed a significant response of *SlMYB78-like* to ABA. Further investigation demonstrated that, in the ABA sensitivity assay, no significant difference was observed between the wild-type and transgenic seedlings under 0 μM and 3 μM ABA. However, when the ABA concentration was increased to 6 μM, the root length of the wild-type seedlings was significantly inhibited, while the inhibition in transgenic lines was notably less pronounced, although no significant difference in hypocotyl length was observed under varying concentrations (Figure 2A–C). Given the reduced sensitivity of transgenic tomato seedlings to ABA, the qRT-PCR assay indicated that the transcript level of the *SlCYP707-A2* gene, which encodes a protein related to ABA degradation, was promoted evidently in transgenic lines. Conversely, the expression of *SlPYL2,* which encodes an ABA receptor protein; *SlABI3* and *SlABI5,* which encode proteins involved in the ABA response; and *SlNCED1* and *SlNCED2,* which encode proteins involved in the ABA biosynthesis was down-regulated significantly in transgenic lines compared with the wild type (Figure 2D–I). These results suggested that the down-regulation of ABA receptor and response genes, coupled with the up-regulation of ABA degradation-related genes, may contribute to the reduced sensitivity of transgenic lines to ABA treatment.

### 2.4. Silencing of SlMYB78-like Reduced Mannitol and NaCl Tolerance of Tomato Seedlings

**Figure 3:**
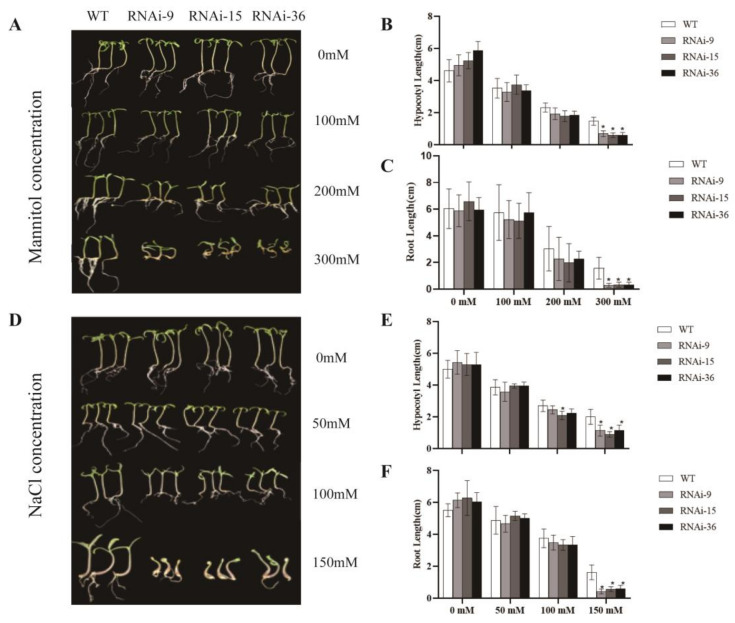
(**A**,**D**) Growth of WT and SlMYB78-like-RNAi seedlings under different concentrations of mannitol and NaCl treatment. (**B**,**E**) Hypocotyl length of WT and *SlMYB78-like*-RNAi seedlings under mannitol and NaCl treatments. (**C**,**F**) Root length of WT and *SlMYB78-like*-RNAi seedlings under mannitol and NaCl treatments All data are presented as means (±SE) from three independent biological replicates. Asterisks above each column indicate significant differences between WT and transgenic lines (* *p* < 0.05).

To investigate the role of *SlMYB78-like* in drought stress response, we assessed the drought tolerance of transgenic and wild-type tomato seedlings using mannitol. Prior to mannitol treatment, no significant morphological differences were observed between the wild-type and transgenic seedlings in plant morphology. However, as the mannitol concentration increased, the growth of both the transgenic and wild-type seedlings was inhibited, with the transgenic seedlings exhibiting more severe growth inhibition compared to the wild-type seedlings. Notably, at a mannitol concentration of 300 mM, transgenic seedlings were nearly incapable of normal growth (Figure 3A). The results demonstrated that both the hypocotyl length and root length of the transgenic seedlings were significantly shorter than those of the wild-type seedlings, with the most pronounced differences observed at the 300 mM concentration (Figure 3B,C). These findings indicated that silencing *SlMYB78-like* diminished drought tolerance in seedlings, thereby compromising their resistance to drought stress.

To further investigate the role of SlMYB78-like in salt tolerance, wild-type and transgenic seeds were germinated on media containing different concentrations of sodium chloride. In the absence of NaCl treatment, no significant differences were observed between the wild-type and transgenic tomato seedlings. However, as the sodium chloride concentration increased, the growth of both transgenic and wild-type seedlings was inhibited, with the transgenic seedlings exhibiting more severe growth inhibition compared to the wild-type seedlings. At 150 mM sodium chloride, transgenic seedlings exhibited a nearly complete inability to grow normally (Figure 3D). A statistical analysis of the stem and root lengths under different salt concentrations revealed that both parameters were significantly reduced in the transgenic seedlings compared to the wild-type seedlings at concentrations exceeding 100 mM, with the most pronounced differences observed at 150 mM (Figure 3E,F). These results indicated that silencing of *SlMYB78-like* compromised salt tolerance in seedlings.

### 2.5. Silencing of SlMYB78-like Inhibited Tomato Plant Resistance to Drought and Salt Stress

**Figure 4:**
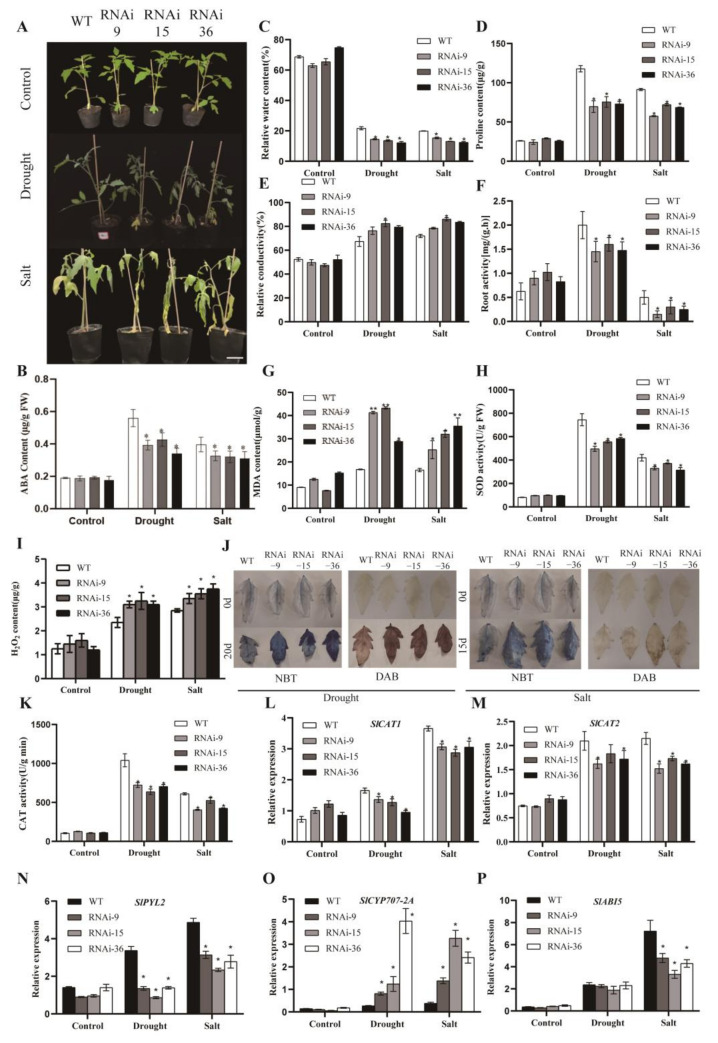
(**A**) Growth characteristics of WT and *SlMYB78-like*-RNAi tomato plants before and after drought/salt treatment. (**B**) Measurement of endogenous ABA in plants before and after treatment. (**C**–**I**) Comparison of relative water content (RWC) (**C**), proline content (**D**), relative conductivity (**E**), root activity (**F**), malondialdehyde (MDA) content (**G**), SOD activity (**H**), H_2_O_2_ content (**I**), DAB and NBT staining (**J**), and CAT activity (**K**) between WT and *SlMYB78-like*-RNAi plants after drought and salt stress. (**L**–**P**) Transcript level of *SlCAT1*, *SlCAT2*, *SlPYL2*, *SlCYP707-A2*, and *SlABI5* before and after treatment. All data are presented as means (±SE) from three independent biological replicates. Asterisks above each column indicate significant differences between WT and transgenic lines (* *p* < 0.05, ** *p* < 0.01).

To further evaluate the function of *SlMYB78-like* in abiotic stress response, we performed drought and salt stress experiments employing WT and *SlMYB78-like*-RNAi lines at 5 weeks of age. Figure 4A depicted the appearance of tomato seedlings before and after treatments. Following drought stress, WT plants showed only minor wilting, while *SlMYB78-like*-RNAi plants exhibited substantial wilting symptoms on their leaves. After salt treatment, many leaves of WT plants remained relatively intact and green, whereas *SlMYB78-like*-RNAi plants showed severe yellowing, wilting, and even necrosis. Further, we discovered that *SlMYB78-like*-RNAi lines had lower accumulation of endogenous ABA (Figure 4B), relative water content (Figure 4C), proline content (Figure 4D), root activity (Figure 4F), SOD (superoxide dismutase) activity (Figure 4H), and CAT (catalase) activity (Figure 4K) compared to the wild type, while the *SlMYB78-like*-RNAi lines processed higher relative conductivity (Figure 4E), MDA (malondialdehyde) content (Figure 4G), and H_2_O_2_ content (Figure 4I) compared to the wild type. Moreover, DAB and NBT staining also revealed that the transgenic lines accumulated more H_2_O_2_ and superoxide anion compared to the wild type after drought and salt stresses (Figure 4J). Subsequently, the qRT-PCR results indicated that the expression level of *SlCAT1/2*, *SlPYL2*, and *SlABI5* was inhibited significantly in the transgenic lines while *SlCYP707-A2* showed the opposite trend (Figure 4L–P).

### 2.6. Silencing of SlMYB78-like Inhibited the Accumulation of Chlorophyll under Stress Conditions

**Figure 5:**
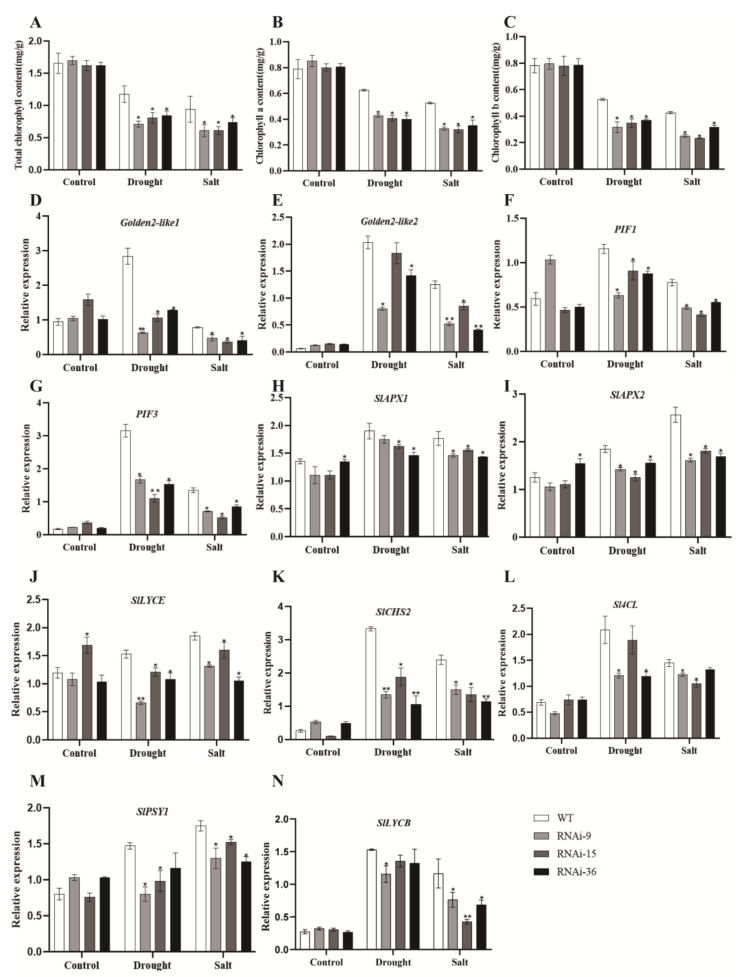
(**A**–**C**) Total chlorophyll, chlorophyll a, and chlorophyll b content in the leaves of SlMYB78-like silenced lines and WT plants under drought and salt stress. (**D**–**G**) The expression levels of *SlGolden2-like1*, *SlGolden2-like2*, *SlPIF1*, and *SlPIF3,* which were closely related to chlorophyll biosynthesis and photosynthesis in the leaves of the *SlMYB78-like* silenced lines and WT plants under drought and salt stress. (**H**–**N**) Expression levels of *SlAPX1*/*SlAPX2*, *SlLYCE*, *SlCHS2*, *Sl4CL*, *SlPSY1*, and *SlLYCB* in the leaves of *SlMYB78-like* silenced lines and WT plants under drought and salt stress. The experimental results were biologically repeated three times. All data are the mean (±SE) of 3 independent biological replicates (* *p* < 0.05, ** *p* < 0.01).

Chlorophyll content serves as a crucial important physiological indicator for measuring plant resistance to adverse conditions. Under stress conditions, plants exhibit a significant reduction in chlorophyll accumulation. This study revealed that prior to drought treatment, there were no significant differences in the contents of chlorophyll a, chlorophyll b, and total chlorophyll between the wild-type and transgenic lines. However, after drought treatment, the transgenic lines displayed significantly lower levels of chlorophyll a, chlorophyll b, and total chlorophyll in their leaves compared to the wild-type (Figure 5A–C). Concurrently, the transcription levels of *Golden2-like1* and *Golden2-like2*, which are photosynthesis-related nuclear genes essential for driving chloroplast biogenesis, were significantly down-regulated (Figure 5D,E). Furthermore, transcription levels of the phytochrome factors *PIF1*/*PIF3* were also significantly down-regulated (Figure 5F,G). These findings demonstrated that silencing of *SlMYB78-like* reduced the chlorophyll contents of transgenic plants by affecting the transcription level of genes involved in chlorophyll synthesis, thereby weakening photosynthesis under drought and salt stress and consequently diminishing the plant’s stress resistance. In addition, the qRT-PCR results revealed that the mRNA abundance of *SlAPX1*/*SlAPX2* and *SlLYCE*, *SlCHS2*, *Sl4CL*, *SlPSY1*, and *SlLYCB* were down-regulated evidently in *SlMYB78-like*-RNAi lines compared to the wild-type after stress exposures (Figure 5H–N).

### 2.7. Phenotypic Observation After Rehydration and Detection of Physiological Indicators Related to Cell Damage

**Figure 6:**
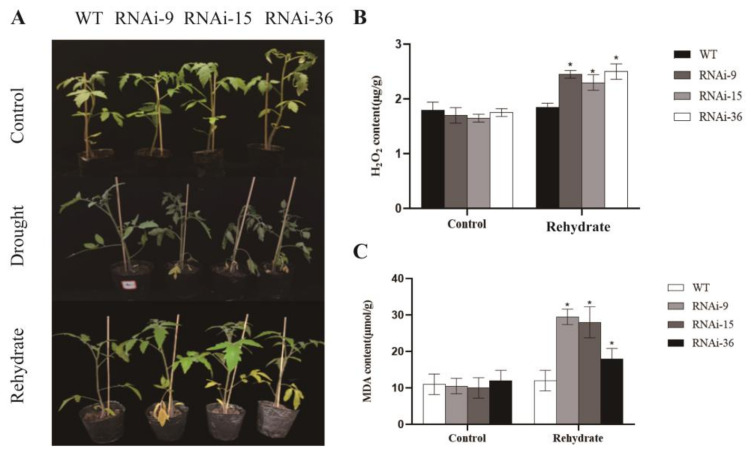
(**A**) Phenotypic diagram of WT and SlMYB78-like-RNAi before and after rehydration treatment; (**B**) determination of H_2_O_2_ content of WT and SlMYB78-like-RNAi before and after rehydration treatment; (**C**) determination of WT and SlMYB78-like-RNAi MDA content before and after rehydration treatment. Scale bar is 1 cm. WT: wild-type tomato; ±SE, standard deviation. Asterisks indicate significant differences between the silenced strain and the wild-type strain after drought treatment (*t* test, * *p* < 0.05).

A 2-day rehydration experiment was conducted using wild-type and transgenic lines after drought as experimental materials, with plants grown under normal conditions for the same number of days as controls. The results showed that after rehydration treatment, there were no significant differences in plant appearance between the wild-type plants and the controls. Although the transgenic tomatoes partially recovered from the wilting state, the overall degree of recovery was not as significant as that of the wild-type plants (Figure 6A). Simultaneously, in order to explore whether there were significant differences in indicators related to cell damage during the rehydration process, we tested the hydrogen peroxide (H_2_O_2_) content and malondialdehyde content in leaves. The results demonstrated that under normal growth conditions, there were no significant differences between the transgenic plants and the wild-type plants. However, after rehydration, the levels of hydrogen peroxide (H_2_O_2_) and malondialdehyde (MDA) contents in the wild-type tomatoes plants almost returned to normal levels, while those in the transgenic plants did not return to normal levels (Figure 6B,C). These results indicated that the transgenic lines had poorer recovery ability than the wild-type after rewatering, further demonstrating that the *SlMYB78-like* silenced lines processed reduced resistance to drought stress.

### 2.8. SlMYB78-like Inhibited the Transcriptional Activity of SlCYP707-A2

**Figure 7:**
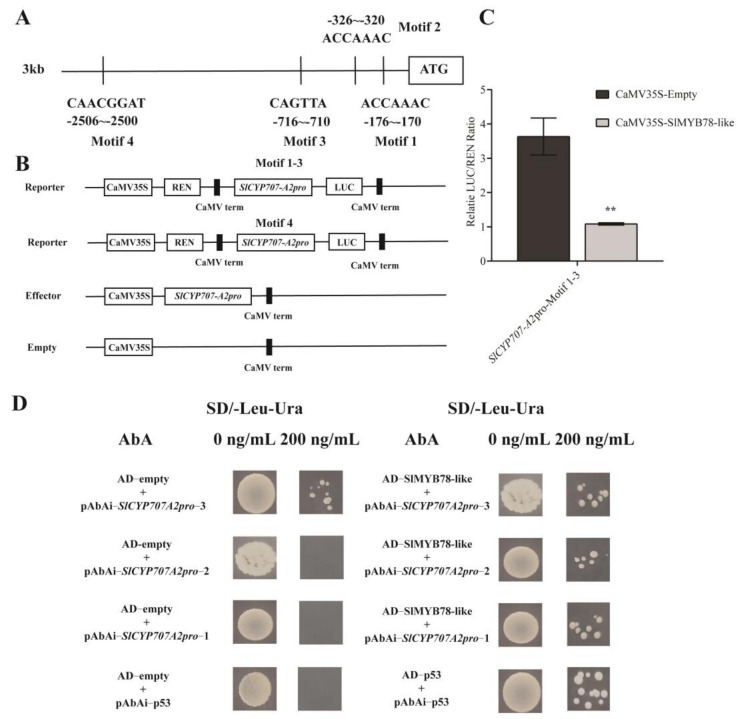
The regulation of SlMYB78-like to SlCYP707-A2. (**A**) The distribution of the binding site in the *SlCYP707*-*A2* promoter. (**B**) A schematic diagram of the vector construction of the effector and reporter. (**C**) The results of the Dual-LUC experiment demonstrated that SlMYB78-like enabled the repression of the transcript of *SlCYP707-A2*. Data are means ± SD of three biological replicates. Statistically significant differences were determined using Student’s *t*-test (** *p* < 0.01). (**D**) Y1H assay for identification of binding of SlMYB78-like to the promoter of *SlCYP707-A2*.

Based on the alteration in ABA content and transcript level of the related genes, we observed that the mRNA abundance of *SlCYP707-A2*, which encodes a protein involved in ABA metabolism, was up-regulated significantly. It functions in catalyzing the hydroxylation at the 8′-methyl group of ABA, leading to ABA being inactive. An analysis revealed the presence of 4 *cis*-acting motifs in the 3kb promoter (Figure 7A). A Dual-luciferase assay demonstrated that SlMYB78-like targeted to the motif 1–3 region, but not motif 4 (Figure 7B,C). In order to identify which motif it binds to, we cloned three motifs separately into pAbAi and performed Y1H with pGADT7-SlMYB78-like. The results exhibited that SlMYB78-like combined with motif 1 and motif 2 (Figure 7D). These findings indicated that SlMYB78-like directly targeted *SlCYP707-A2* directly and inhibited its transcription.

### 2.9. SlMYB78-like Physically Interacted with SlDREB3

**Figure 8:**
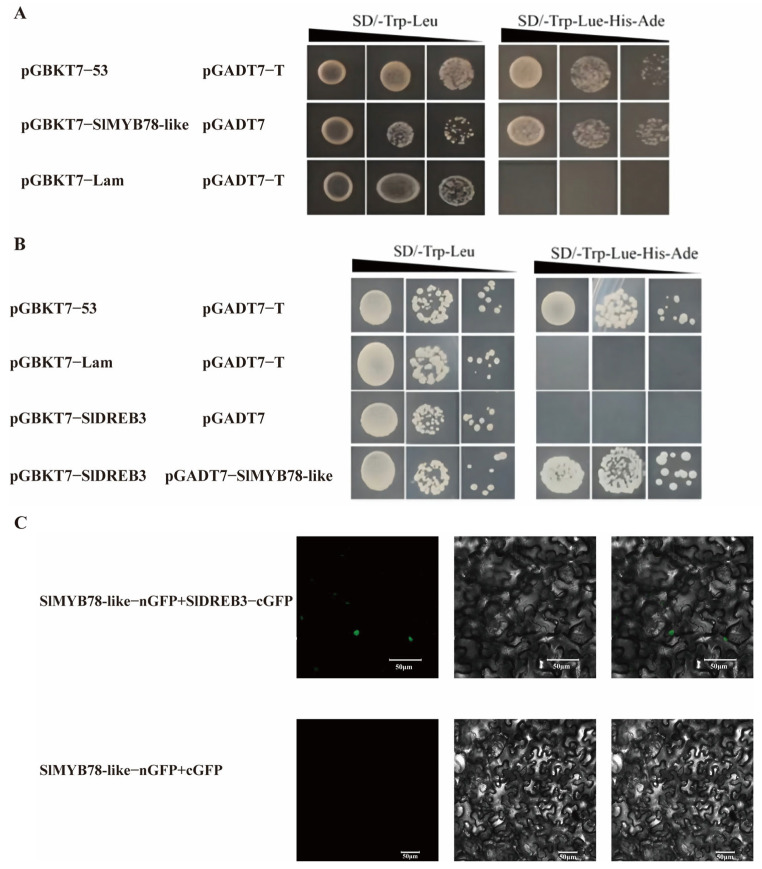
The identification of protein–protein interaction between SlMYB78-like and SlDREB3. (**A**) Transcription activity analysis of SlMYB78-like and the results showed that it had self-activation ability. pGBKT7-53 and pGADT7-T were the positive control, while pGBKT7-Lam and pGADT7-T were the negative control. (**B**) SlDREB3 was cloned into pGBKT7, while SlMYB78-like was cloned into pGADT7. It showed that SlDREB3 did not self-activate, and the protein interaction between them was characterized. (**C**) The BiFC assay was employed to verify the interaction of SlMYB78-like and SlDREB3. Bar = 50 μm.

SlMYB78-like was identified as a typical R2R3 transcription factor belonging to the MYB family. Previous research has demonstrated that a key mechanism through which R2R3 transcription factors exert their regulatory role is mediated by protein–protein interactions. In the present study, we generated a *SlMYB78-like*-pGBKT7 fused vector and transformed it into yeast competent cells. The transformed cells exhibited normal growth when cultured on SD/-Trp-Leu-His-Ade medium, indicating that SlMYB78-like processed transcriptional activation activity (Figure 8A). In addition, we confirmed that SlDREB3 lacked self-activation properties. To enhance the accuracy of the experiment, we cloned *SlMYB78-like* into the pGADT7 to minimize false-positive results. Furthermore, the Y2H assay provided positive evidence for the physical interaction between SlMYB78-like and SlDREB3 (Figure 8B).

To further investigate the protein interaction between SlMYB78-like and SlDREB3, a bimolecular fluorescence complementation (BiFC) assay was conducted. The fused expression vectors SlMYB78-like-nGFP and SlDREB3-cGFP were constructed and transformed into *A. tumefaciens* strain GV3101. Transient expression experiments were performed, refering to the established protocols from previous research. The BiFC assay results confirmed the protein–protein interaction between SlMYB78-like and SlDREB3 (Figure 8C), illustrating that SlMYB78-like potentially functioned in the ABA signaling pathway through its interaction with SlDREB3.

## 3. Discussion

Drought and high salinity are prevalent abiotic stresses in the natural environment. Plants have developed intricate defense mechanisms at the molecular, physiological, and biochemical levels to adapt to these adverse conditions [33]. Herein, we characterized the function of *SlMYB78-like* in tomato in response to abiotic stresses by RNAi, further exploring its comprehensive regulatory mechanism.

The analysis of *cis*-acting elements in the promoter region and the induction of drought/salt stress implied that *SlMYB78-like* potentially contributed to the stress response under these conditions. Furthermore, in the present study, the seedlings and 5-week-old plants of *SlMYB78-like*-RNAi lines exhibited reduced tolerance to both drought and salt stresses. In a previous study, the overexpression of *FtMYB22* in *Arabidopsis* from Tartary buckwheat (*Fagopyrum tataricum* Gaertn.) resulted in reduced tolerance to salt and water-deficit stresses, accompanied by higher accumulation of MDA, H_2_O_2_, and O_2_^−^ [34]. Similarly, the down-regulation of *GhMYB36* via VIGS (Virus-Induced Gene Silence) resulted in decreased tolerance to drought stress along with an increased accumulation of MDA [35]. The overexpression of strawberry *FvMYB24* in *Arabidopsis* generated an enhanced resistance to salt stress, displaying higher activities of superoxide dismutase (SOD), peroxidase (POD), and catalase (CAT) and reduced accumulation of MDA and H_2_O_2_, demonstrating the positive function of *FvMYB24* in salt stress [36]. Under natural conditions, stressors induce the accumulation of reactive oxygen species (ROS), H_2_O_2_, and MDA, which cause oxidative damage to cells, thereby threatening plant growth and crop yield [37,38]. Plants mitigate ROS damage through the regulation of antioxidant enzyme systems, which play crucial roles in stress tolerance; higher activities of these enzymes correspond to enhanced stress resistance in plants [39]. In the present study, the silencing of *SlMYB78-like* exhibited reduced tolerance to drought and salt stresses, accompanied by a decreased content of proline and the increased accumulation of MDA and H_2_O_2_. Meanwhile, DAB and NBT staining also illustrated increased accumulation of H_2_O_2_ and superoxide anion. These results indicated that the silencing of *SlMYB78-like* led to increased accumulation of peroxide, causing severe damage to cell membranes and further exhibiting a reduced tolerance to drought and salt stresses. In addition, the activities of POD and SOD in the leaves of the *SlMYB78-like*-RNAi lines after the drought and salt treatments were lower than those in the wild-type (WT) plants, suggesting that the tolerance of the *SlMYB78-like*-RNAi lines was inhibited significantly. Moreover, when exposed to salt and drought stresses, the *SlMYB78-like*-RNAi lines exhibited lower water storage, further demonstrating that *SlMYB78-like* functioned as a positive regulator in abiotic stress response.

Generally, plants accumulate organic matter via photosynthesis, promoting growth and development, and the content of chlorophyll was positively correlated with photosynthetic rate within a certain range. Stresses accelerate chlorophyll degradation, making chlorophyll content a crucial physiological indicator for assessing plant stress resistance [40,41]. Drought stress significantly influences light energy absorption and photosynthesis rate in plants, affecting carbon assimilation by inhibiting electron transport and phosphorylation activity [42,43]. Research indicated that *Golden2-like1* and *Golden2-like2* are indispensable nuclear genes for chloroplast biogenesis and photosynthesis [44,45]. Moreover, reduced transcript levels of phytochrome-interacting factors *PIF1* and *PIF3* decrease photosynthetic activity [46,47]. In this study, we measured the chlorophyll content in *SlMYB78-like*-RNAi and WT plants under drought and high salinity stress. The results indicated that the synthesis of chlorophyll in the *SlMYB78-like*-RNAi lines was repressed under stress conditions compared to the wild type. Additionally, the transcriptional levels of *Golden2-like1*, *Golden2-like2*, *PIF1*, and *PIF3* in the *SlMYB78-like*-RNAi lines were 0.5 to 1.5 times lower than those in the WT plants after salt and drought stresses. The overexpression of the oil palm R2R3-MYB subfamily genes *EgMYB111* and *EgMYB157* endowed the enhanced tolerance of transgenic *Arabidopsis* to abiotic stresses, along with the increased accumulation of photosynthetic pigments and net photosynthetic rate [48]. Similarly, the overexpression of *FtMYB13* from Tartary buckwheat in *Arabidopsis* improved the tolerance to drought/salt and the photosynthetic efficiency [49]. In soybean (*Glycine max* L.), *GmMYB68* positively regulates the response to soil salinity, with overexpressed-*GmMYB68* lines exhibiting enhanced tolerance to salt and photosynthetic rates [50]. In our study, stress decreased the content of chlorophyll in the *SlMYB78-like*-RNAi lines compared with the wild type and together with the down-regulation of transcript level of genes involved in chlorophyll biosynthesis and photosynthesis. These findings illustrated the *SlMYB78-like*’s positive function in abiotic stress response.

ABA is a crucial plant hormone which mediates plant responses to environmental stresses. In *Arabidopsis*, DIV2, which was identified as a MYB transcription factor, was induced markedly by ABA, and the *div2* mutant exhibited promoted tolerance via ABA signaling [51]. Transgenic *Arabidopsis* lines overexpressing *AcoMYB4*, a MYB transcription factor from *Ananas comosus* L., displayed increased sensitivity to abiotic stress, possibly via inhibiting the transcript level of *ABA1/2* and *ABI5*, which are involved in ABA synthesis and signal transduction [52]. The overexpression of *ZmMYB3R* enhanced the tolerance of the transgenic *Arabidopsis* lines to abiotic stress and altered the ABA sensitivity, along with increased accumulation of endogenous ABA, illustrating its positive role in an ABA-dependent pathway [53]. In the present study, the transcript level of *SlMYB78-like* was induced evidently by exogenous ABA, and the seedlings of the *SlMYB78-like*-RNAi lines exhibited reduced sensitivity to ABA, along with inhibition of the transcript level of genes involved in ABA signaling transduction and biosynthesis, while the transcript level of the degradation gene was increased. ABA sensitivity is the plant’s capacity to respond to ABA signals, influencing the vital processes such as seed dormancy, germination, and adaptation to environmental stress. ABA sensitivity is determined by the perception and transduction of the ABA signal, and mutations in components of this pathway can reduce sensitivity and impair stress responses [54]. Notably, the effect of exogenous ABA on root elongation was biphasic, with high concentrations of ABA inhibiting root growth and low concentrations playing a positive role [55]. In the present study, as the ABA concentration increased, the root length of both the wild-type and transgenic lines was significantly inhibited. However, the root length of the RNAi lines was evidently longer than that of the wild type, illustrating that the inhibitory effect of ABA on root development was weakened in the RNAi lines. Combined with the changes in the transcript-level of related genes (*ABI3* and *ABI5*), we proposed that the ABA response was altered in the *SlMYB78-like*-RNAi lines and that silencing of *SlMYB78-like* altered the ABA sensitivity. Moreover, when exposed to salt and drought stresses, the transgenic lines’ plants accumulated less endogenous ABA compared to the wild type. These results demonstrated that *SlMYB78-like* was closely correlated with the tolerance to salt and drought stresses via the ABA pathway. Moreover, we found that the mRNA abundance of *SlCYP707A2*, which encodes ABA 8′-hydroxylase, a key enzyme in the ABA metabolism pathway [56], was inhibited significantly after stresses in the transgenic lines compared to the wild-type. For *SlCYP707-A2,* previous studies have reported increased ABA levels in the dry and hydrated seeds of *cyp707a2* mutants [57]. Additionally, the expression of *CYP707A1* and *CYP707A2* is highly induced under drought stress in *hda9-1*, *hda9-2*, *abi4*, and *hda9-1 abi4* mutants, with a complex of *HDA9* and *ABI4* repressing their expression by binding to their promoters [58]. Moreover, many members of the MYB transcription factor family members are involved in plant responses to abiotic stresses such as drought and salt stresses through the ABA pathway [59,60,61]. Combining previous research with our experimental results, we hypothesized that the *SlMYB78-like* gene may positively regulate plant responses to drought and salt stresses via the ABA pathway. To explore the regulatory mechanisms of the *SlMYB78-like* under drought and salt stress, we conducted Dual-LUC, Y1H, Y2H, and BiFC assays. We first identified *SlCYP707-A2*, a gene significantly up-regulated in the silenced transgenic lines, as a potential downstream target regulated by SlMYB78-like. Using the Dual-LUC reporter system, we found that the SlMYB78-like bound to the *SlCYP707-A2* promoter and inhibited its transcriptional activity. Further validation through yeast one-hybrid assays showed that SlMYB78-like could bind to segments of the *SlCYP707-A2* promoter containing the CAGTTA motif. Our findings suggested that the silencing of the *SlMYB78-like* alleviated the repression of the expression of the ABA degradation gene *SlCYP707-A2*, enhancing the degradation of endogenous ABA. Consequently, *SlMYB78-like* transgenic plants were less sensitive to ABA, resulting in reduced resistance to drought and salt stress.

In this study, the silencing of *SlMYB78-like* reduced plant tolerance to drought and salt stresses with an inhibited accumulation of endogenous ABA, indicating that *SlMYB78-like* functioned through the ABA pathway. For further exploring the mechanism of SlMYB78-like in the ABA pathway, we performed a Y2H assay and finally screened a transcription factor, SlDREB3. The BiFC experiment also confirmed their interaction. Previous research has been identified *SlDREB3* as an abiotic stress-responsive gene [62]. Moreover, as a dehydration response factor, the ectopic expression of *SlDREB3* influenced plant growth and root architecture through the ABA signaling pathway [63]. Moreover, *SlDREB3* was involved in abiotic stress responses via the ABA pathway. For instance, the overexpression of *SlDREB3* promoted the transcript levels of *SlLEAs*, enhancing the cold stress resistance of transgenic tomatoes [64]. These studies illustrated that SlDREB3 functioned in abiotic stress through ABA response. Our current findings revealed a physical interaction between SlMYB78-like and SlDREB3, suggesting that SlMYB78-like possibly played a role in ABA response by interacting with SlDREB3, consequently affecting plant tolerance to drought and salt stress.

**Figure 9:**
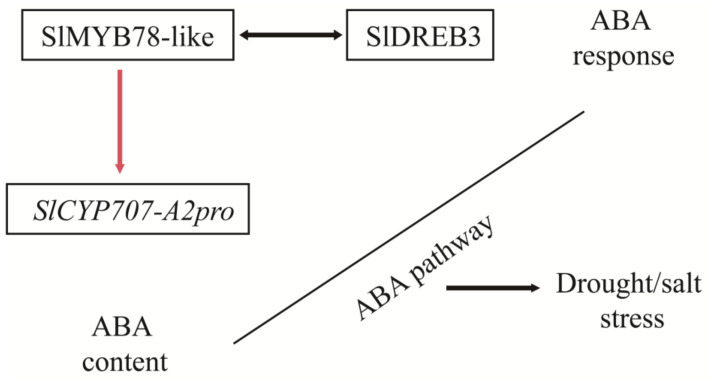
Schematic of *SlMYB78-like* involved in drought and salt stresses via the ABA pathway. The double-headed arrows indicate the protein interaction between SlMYB78-like and SlDREB3, involved in ABA response. The red arrows indicate that SlMYB78-like inhibited the transcription of *SlCYP707-A2*, influencing the content of ABA. Black line and arrow indicated that SlMYB78-like positively governed the tolerance of plant to drought and salt via the ABA pathway, including ABA response and ABA content.

In conclusion, this study characterized the function of *SlMYB78-like* in drought and salt stress response and proposed a regulation model (Figure 9). The silencing of *SlMYB78-like* in tomato resulted in a reduced tolerance to drought and salt stress, accompanied by repressed ABA content. Moreover, SlMYB78-like physically interacted with SlDREB3, further participating in the ABA response. In addition, SlMYB78-like directly targeted and suppressed the transcription of *SlCYP707-A2* directly, altering the accumulation of the plant hormone ABA. Collectively, these findings demonstrated that *SlMYB78-like* functioned as a positive regulator in drought and salt stress response via the ABA pathway.

## 4. Materials and Methods

### 4.1. Construction of SlMYB78-like-RNAi Vector and Plant Transformation

A 551 bp fragment of *SlMYB78-like* (551 bp), located in the non-conserved domain to avoid silencing other genes, was cloned into pBIN19 to construct the *SlMYB78-like*-RNAi vector, following the method described in a previous report [28]. Subsequently, the completed *SlMYB78-like*-RNAi vector was introduced into *Agrobacterium LBA4404* and then transformed into wild-type tomato using a method previously described [65]. Positive transgenic lines were selected based on their resistance to kanamycin (50 mg/L) and confirmed by PCR and qRT-PCR using specific primers (Table S1).

### 4.2. Total RNA Extraction and Quantitative Real-Time PCR (qRT–PCR) Analysis

Total RNA was extracted using Trizol reagent (Invitrogen, Shanghai, China) following the established protocols from previous studies. The CFX connect real-time system (Bio-Rad, Hercules, CA, USA) was used to perform quantitative reverse transcription PCR (qRT-PCR) following the methodology detailed in prior research. The relative gene expression levels were analyzed using the 2^−ΔΔCT^ method, with the gene expression was normalized with the *SlCAC* gene (*Solyc08g006960*) as the internal standard [66,67]. The experiment included three independent biological replicates performed for each assay. All primer sequences used in this study are listed in Table S1.

### 4.3. Sample Collection in Different Experiments

In the present study, *Solanum lycopersicum* Mill. cv. Ailsa Craig, AC^++^ was used as the wild type. All the tested plants including the wild type and transgenic lines were grown in soil under a 16 h light and 8 h dark cycle at 28 °C during the light phase and 18 °C during the dark phase.

For tissue expression pattern analysis, samples were collected from various plant parts, including Rt (roots), St (stems), YL (young leaves), ML (mature leaves), SL (senescent leaves), Fl (flowers) and fruit samples at IMG (immature green), MG (mature green), B (break, which fruit start to ripening), B + 4 (4 days after break), and B + 7 (7 days after break). All collected samples were frozen in liquid nitrogen immediately for further analysis.

Five-week-old plants grown in soil were employed for exogenous ABA response analysis. The leaf surfaces were sprayed uniformly with a 100 μM ABA solution and then incubated at 28 °C. Leaf samples from the same sprayed location were collected at 0 h, 1 h, 2 h, 4 h, 8 h, 12 h, and 24 h post-treatment and frozen in liquid nitrogen for future analysis.

Five-week-old plants grown in soil were subjected to drought and salt stress. The soil attached to the roots was washed off and carefully removed, and the seedlings were placed at 28 °C for drought treatment. For salt stress treatment, the roots were irrigated with 300 mM sodium chloride solution, ensuring complete submersion of the roots. The excess sodium chloride solution was then drained. Leaf samples were collected from the same location at 0, 1, 2, 4, 8, 12, and 24 h post-treatment. The samples were immediately flash-frozen in liquid nitrogen and stored at −80 °C for further analysis.

### 4.4. The Effect of Mannitol, Salt, and ABA on Tomato Seedlings

After surface sterilization, the seeds of the wild-type and transgenic lines were sown on MS medium containing varying concentrations of mannitol (0 mM, 100 mM, 200 mM, and 300 mM), sodium chloride (0 mM, 50 mM, 100 mM, and 150 mM), and ABA (0 μM, 3 μM, and 6 μM). Following seven days of cultivation, the length of the roots and hypocotyls were measured, and the samples were collected and stored in −80 °C for further analyze. The experiment was repeated three times, with a minimum of 30 seedlings measured in each replicate.

### 4.5. Drought and Salt Tolerance Experiments in Tomato Plants

For drought stress treatment, the 5-week-old wild-type and transgenic plants were grown in soil under well-watered conditions. At the start of the stress treatment (designated as “0 d”), watering was ceased, and the plants were subsequently maintained under identical environmental conditions.

For salt stress treatment, 300 mM sodium chloride solution was added to the soil in which the plants were growing, and the plants were irrigated with the same salt solution every 2 days. The growth status of the plants was observed and recorded throughout the experiment. Leaf samples were collected from the same location at the beginning of stress treatment (“0 d”) and after 15 days of stress for RNA extraction and measurements of related indices. The experiment was conducted in triplicate to ensure reproducibility.

### 4.6. Measurement of Relative Water Content, Water Loss Rate, Relative Conductivity, Chlorophyll Contents, Proline, Hydrogen Peroxide, and Malondialdehyde

For the determination of relative water content (RWC), the fresh weight (FW), dry weight (DW), and saturated weight (SW), the leaves from soil-grown plants were measured for these parameters. RWC was calculated using the following formula:

RWC = [(FW − DW)/(SW − DW)] × 100%

For the water loss rate determination, the leaves (30 pieces) of both the WT and the *SlMYB78-like*-RNAi lines grown in soil were collected and placed on dry filter paper. The leaves were weighed at 30 min intervals for a total of 240 min, and the relative water loss rates were calculated based on these measurements.

Relative conductivity was determined by punching 25 leaves from each group of soil-grown plants and placing them in a tube containing 20 mL of double-distilled water (ddH_2_O). After a 12 h incubation period, the initial conductivity (R1) was measured. The tube was then heated in a boiling water bath for 30 min and then cooled to room temperature, and the final conductivity (R2) was recorded.

Relative conductivity = R1/R2 × 100%

The ethanol extraction method was employed to determine the chlorophyll content. The following equations were used to calculate the total chlorophyll, chlorophyll a, and chlorophyll b concentrations, where A_663_ and A_645_ represent absorbance values at 663 nm and 645 nm, respectively [68]:

Total chlorophyll (mg/L FW) = 8.02 × A_663_ + 20.2 × A_645_

Chlorophyll a (mg/L FW) = 12.7 × A_663_ − 2.69 × A_645_

Chlorophyll b (mg/L FW) = 22.9 × A_645_ − 4.677 × A_663_

For proline content determination, a 0.2 g leaf sample was ground into powder using liquid nitrogen and mixed with 1.3 mL of 3% sulfosalicylic acid. The mixture was heated in a boiling water bath for 10 min, cooled to room temperature, and centrifuged at 12,000 rpm for 15 min. In a 10mL centrifuge tube, 1 mL of glacial acetic acid and 1mL of indanone were added to 1mL of the supernatant. The mixture was then heated in a boiling water bath for 30 min. After cooling, 3mL of toluene was added, and the mixed solution was inverted and allowed to stand for 10 min before measuring the absorbance at 520 nm (OD_520_). The proline content (µg/g) = (C × Vt)/(W × Vs), where C is the proline concentration on the standard curve (µg/mL), Vt is the total volume of the sample extract (mL), W is the fresh weight of the leaves (g), and Vs is the volume of the sample extract used to determine absorbance (mL) [69].

The content of hydrogen peroxide (µg/g) was calculated via (C × Vt)/(W × Vs). C represents the H_2_O_2_ concentration determined based on the standard curve (unit: µg/L), Vt represents the total volume of the sample extract (in milliliters), W represents the fresh weight of the tested leaves (g), Vs represents the volume of sample extract used for measuring absorbance [70].

The malondialdehyde content was calculated using the following formula (µmol/g): [6.45 × (OD_532_ − OD_600_) − 0.56 × OD_450_] × Vt/(Vs × W). OD_532_, OD_600_, and OD_450_ means optical densities at 532nm, 600nm, and 450nm. Vt denotes the total volume of the sample extract, measured in milliliters; Vs signifies the volume of extract utilized during the measurement process, and W corresponds to the net weight of the sample [71].

For root activity, the TTC (triphenyltetrazole chloride) reduction assay was employed, following the methodology described by [72].

### 4.7. Enzyme Activity Assay

The collected leaf samples were frozen in liquid nitrogen and ground into a fine powder. A 1.8 mL PBS buffer was added to the powder, and the mixture was centrifuged at 16,000× *g* at 4 °C for 20 min. The resulting supernatant was collected for the further determination.

To measure CAT activity, the samples were mixed with H_2_O_2_, H_2_O, and enzyme solution. The rate of decrease in OD_240_ was then rapidly measured. CAT activity(µg/min): (∆A_240_ × Vt)/(W × Vs × 0.01 × t), where ∆A_240_ represents the change in OD_240_ per unit of time, Vt is the total reaction volume (milliliters), Vs is the volume of enzyme solution tested (milliliters), W is the fresh weight (grams), and t is the reaction time (minutes) [73].

For POD activity, 20 μL of the extract was added to 180 μL of the POD reaction buffer, which was prepared from PBS buffer, 10 mM hydrogen peroxide (H_2_O_2_), and 10 mM guaiacol. The absorbance at A_470_ was measured, and the change in absorbance per unit time was recorded. POD activity (μ/g min) = (ΔA_470_ × Vt)/(W × Vs × 0.01 × t). Vt, Vs, W, and t represent the same parameters as in the CAT method [74].

For SOD activity, 50 μL of the extract was added to an SOD reaction buffer consisting of 220 mmol/L Met, 1.25 mmol/L NBT, 0.033 mmol/L riboflavin, and PBS buffer. Four control groups were established, with the enzyme solution replaced by the buffer solution. One control group was placed in the dark, while the other three control groups and the experimental group were exposed to fluorescent light. The dark control group served as a reference for measuring A_560_. SOD activity(µg/FW) = [(Ack − Ae) × V]/(1/2 × W × Vt), where Ack represents the average A_560_ of the three blank groups under light, Ae represents the A_560_ of the experimental group under light, Vt is the total reaction volume (milliliters), V is the volume of enzyme solution tested (milliliters), W is the fresh weight (grams), and t is the reaction time (minutes) [75].

### 4.8. Drought Rehydration Experiment

In order to ascertain the extent of plant recovery following drought treatment, the treated plants were rewatered and cultivated at 28 °C for 48 h. The state of the plants after rehydration was observed, and the H_2_O_2_ and MDA contents were quantified.

### 4.9. Abscisic Acid Content Determination

Leaves from wild-type and transgenic lines, collected before and after exposure to drought and salt stress conditions, were flash-frozen in liquid nitrogen. The samples were subsequently sent to Huakai Biotechnology Co., Ltd. (Weifang, China) for testing.

### 4.10. Dual-Luciferase Assay in Tobacco Leaves

The full-length CDS sequence of *SlMYB78-like* was amplified by PCR using specific primers (Table S1) and cloned into the pGreen II 62-SK vector driven by the cauliflower mosaic virus (CaMV) 35S promoter, serving as an effector. The promoter fragment of *SlCYP707A2* was amplified and cloned into pGreen II 0800-LUC, functioning as a reporter. Firefly luciferase and Renilla luciferase were measured using a Dual-LUC assay kit (Promega, Madison, WI, USA) following the manufacturer’s instructions. The experiment was conducted in triplicate. All primer sequences used in this experiment are listed in Table S1.

### 4.11. Yeast One Hybrid Experiment

The Y1H assays were performed with the yeast strain Y1H Gold. The complete CDS of *SlMYB78-like* was cloned and inserted into the pGADT7 vector. The promoter fragments of *SlCYP707A2* were cloned into the pAbAi vector to generate the pAbAi-*SlCYP707A2*pro construct, which was then transformed into Y1HGold using a PEG/LiAc method. Yeast cells were cultured on the medium lacking Ura (SD/-Ura). Yeast colony PCR was performed to confirm the integration of the positive plasmid integrated into the Y1HGold genome. Subsequently, pGADT7-*SlMYB78-like* was transformed into Y1H Gold-pAbAi-*SlCYP707A2*pro. To identify potential interactions, yeast cells were cultured on media lacking Leu and Ura (SD/-Leu-Ura) supplemented with Aureobasidin A.

### 4.12. Transcriptional Activation Assay and Protein Interaction Experiment

To further analyze the transcriptional activation of SlMYB78-like and SlDREB3, the full-length *SlMYB78-like* and *SlDREB3* CDS sequences were cloned into the pGBKT7 vector. To further analyze the protein–protein interaction between SlMYB78-like and SlDREB3, the full-length *SlMYB78-like* CDS sequence was cloned into the pGADT7 vector. Subsequently, the pGBKT7-*SlMYB78-like* and pGADT7 empty plasmids, the pGBKT7-SlDREB3 and pGADT7 empty plasmids, the pGADT7-SlMYB78-like and pGBKT7-SlDREB3 plasmids, the pGBKT7-53 and pGADT7-T plasmids (positive control), and the pGBKT7-Lam and pGADT7-T plasmids (negative control) were co-transformed into the yeast strain Y2H Gold. The transformed yeasts were grown on SD/-Leu/-Trp medium at 30 °C for 3–5 days. Following this, the positively transformed yeast cells were transferred and dropped onto selective medium (SD/-Leu/-Trp/-Ade/-His medium) and incubated at 30 °C for 3–5 days.

### 4.13. BiFC (Bimolecular Fluorescence Complementation) Assay

The BiFC assay was performed by employing pFGC-cGFP and pFGC-nGFP, with fluorescence signals detected using a confocal laser scanning microscope (Leica, Wetzlar, Germany). The CDS of *SlMYB78-like* and *SlDREB3* were cloned into the vector mentioned above. The complete plasmids were then transferred into the *Agrobacterium tumefaciens* strain GV3101. The experimental group consisted of *SlMYB78-like*-nGFP + *SlDREB3*-cGFP, while *SlMYB78-like*-nGFP + cGFP served as the negative control.

### 4.14. DAB and NBT Staining

For DAB and NBT staining, fresh leaves were collected and placed in DAB and NBT staining solution for 24 h in dark conditions, ensuring that the reaction was fully completed and reducing the interference of light on the color reaction. Subsequently, the leaves were rinsed with double-distilled water and immersed in decolorization solution (ethanol: acetic acid: glycerol = 3:1:1). Decolorization was performed in a boiling water bath for 15 min.

### 4.15. Statistical Analysis

In the present study, each experiment was repeated three times, using 15 plants each time. Moreover, the measurement of physiological indicators and the expression level of each gene was obtained from three independent plants. Statistical analyses were performed using SPSS 26.0 software. The significance level was determined using Student’s *t*-tests at * *p* < 0.05 and ** *p* < 0.01.

## 5. Conclusions

This study identified that the silencing of *SlMYB78-like*, a typical *R2R3-MYB* gene; inhibited the accumulation of endogenous ABA; and reduced plant tolerance to drought and salt stresses. A series of stress-related physiological indicators also confirmed the repressed resistance. Subsequently, this study also identified the protein–protein interaction between SlMYB78-like and SlDREB3, illustrating that SlMYB78-like functioned in ABA response. Furthermore, SlMYB78-like targeted the promoter of *SlCYP707-A2* and inhibited its transcription, repressing ABA degradation and promoting the tolerance to drought and salt stresses. In brief, this study explored the biological role of SlMYB78-like involved in drought and salt stress tolerance via ABA pathway, and the elucidated regulatory mechanism was expected to provide theoretical support for the breeding of tomatoes with enhanced abiotic stress tolerance.

## Data Availability

All relevant data are included within the article and its Supplemental Files.

## Supplementary Materials

The following supporting information can be downloaded at https://www.mdpi.com/article/10.3390/ijms252111449/s1.
