# Supplementary material for: Silencing of SlMYB78-like Reduces the Tolerance to Drought and Salt Stress via the ABA Pathway in Tomato
Source: Int J Mol Sci. 2024 Oct 24;25(21):11449. doi: 10.3390/ijms252111449 (PMC11546358; doi:10.3390/ijms252111449)
Supplement: Supplementary file 1 [file ijms-25-11449-s001.zip › ijms-3213646-supplementary.pdf]

Supplementary Materials

Silencing of *SlMYB78-like* Reduces the Tolerance to Drought and Salt Stress via the ABA Pathway in Tomato

Yu Liu [1,†], Pengyu Guo [1,*,†], Zihan Gao [1], Ting Long [1], Chuanji Xing [2], Jing Li [1], Jing Xue [2], Guoping Chen [1], Qiaoli Xie [1] and Zongli Hu [1,*]

[1] Laboratory of Molecular Biology of Tomato, Bioengineering College, Chongqing University, Room 521, Campus B, 174 Shapingba Main Street, Chongqing 400044, China; 18436099626@163.com (Y.L.); gzhfilm@163.com (Z.G.); longting325112@163.com (T.L.); micy180605@163.com (J.L.); chenguoping@cqu.edu.cn (G.C.); qiaolixie@cqu.edu.cn (Q.X.)

[2] Institute of Grassland, Flowers and Ecology, Beijing Academy of Agriculture and Forestry Sciences, 11 Shuguanghuayuan Middle Road, Haidian, Beijing 100097, China; 18704404680@163.com (C.X.); xuejing@baafs.net.cn (J.X.)

* Correspondence: guopengyu@stu.cqu.edu.cn (P.G.); huzongli@cqu.edu.cn (Z.H.)

† These authors contributed equally to this work.

Table S1. Primer sequence in present study.

Primer	Sequence (5′→3′)
SlCAC-F	CCTCCGTTGTGATGTAACTGG
q-*SlCAC*-R	GAGAAACCGAACACGCAATCC
q-*SlEF1α*-F	TACTGGTGGTTTTGAAGCTG
q-*SlEF1α*-R	AACTTCCTTCACGATTTCATCATA
q-*SlMYB78-like*-F	GTGCTGGTTTGAAGAGAACAGG
q-*SlMYB78-like*-R	TGTTGAGCGATTTTTGACCAAC
q*SlNCED1*-F	CCCGATTTGGTATTCTGGATAAGTA
q*SlNCED1*-R	GAGACGGATTTCGGATAAAACACT
q*SlCAT1*-F	AAATGGGTTGAGTCTTTATCCGA
q*SlCAT1*-R	TCATTGATTTTTCACATTGTAGGCT
q*SlCAT2*-F	TTCTGCCCTTCTATTGTGGTTC
q*SlCAT2*-R	GTGATGAGCACACTTTGGAGC
q*SlCYP707-A2*-F	TCGAAAAAGGATACAATTCGATGCC
q*SlCYP707-A2*-R	CTGCAATTTGTTCGTCAGTGAGTCC
q*SlABI3*-F	TGGGACTTGTCGTGTTTGGAAC
q*SlABI3*-R	CCTCACTTTTACCCCTCGTATCAA
q*SlABI5*-F	CAATACGGGTTGGAAATAGGAA
q*SlABI5-R*	CTTTTAGCTGATTCAATTCTGCC
q*SlPYL2*-F	TCTCTTGTGACGCAACGCAT
q*SlPYL2-R*	GCTACCAACTCCGCCATCA
q*SlGolden2-like1*-F	GAATTTTCCGTAAGCAGTGGTG
q*SlGolden2-like1*-R	CTTCTCCTTGATTTAGGCTCGT
q*SlGolden2-like2*-F	ACAATCGGAGGCGGAGGA
q*SlGolden2-like2-R*	CAAGGAGTGCCTGGTACAAGAG
q*SlPIF3*-F	CCATCAGCCCCAAACTCACT
q*SlPIF3*-R	CTGCCATCTCATTATTGAACTCC
q*SlNCED2*-F	TGGTTTTCATGGGACATTCATTAGC
q*SlNCED2*-R	ATCTCCCTTCTCAACTCCCTATTCC
SlMYB78-like-RNAi-F	CCCAAGCTTGAATTCTTAGCCGAAAGAATACAAGCC
SlMYB78-like-RNAi-R	TGCTCTAGACTCGAGATTTCTCTATATGAATCTCCAACCTC
800-LUC-F	CTGGCGAAAGGGGGATGT
800-LUC-R	TCCAGGAACCAGGGCGTATC

Primer	Sequence (5′→3′)
62-SK-F	TTTCATTTGGAGAGGACAGCC
62-SK-R	TATCGGGAAACTACTCACACATT
62-SK-*SlMYB78-like*-F	CGCGGATCCATGGATTATCATGGTCAGAAAATTG
62-SK-*SlMYB78-like*-R	CCGCTCGAGTCACATGTCATTGTTCAATTGTTGT
LUC-*SlCYP707A2*pro-F	GGGGTACCCCTCGACAATAATAAACCAAAATG
LUC-*SlCYP707A2*pro-R	CCCTCGAGTGAGATATTGGTATCTTTATCGTCT
AD-F	ACGATGCACAGTTGAAGTGAAC
AD-R	TGGAATCACTACAGGGATGTTTAA
BD-F	CCCAAGGGGTTATGCTAGTTAT
BD-R	GCATAGAATAAGTGCGACATCATC
AD-*SlMYB78-like*-F	GGAATTCCATATGATGGATTATCATGGTCAGAAAATTG
AD-*SlMYB78-like*-R	CGCGGATCCTCACATGTCATTGTTCAATTGTTGT
BD-SlMYB78-like-F	GAATTCCATATGATGGATTATCATGGTCAGAAAATTG
BD-SlMYB78-like-R	CGCGGATCCTCACATGTCATTGTTCAATTGTTGT
BD-SlDREB3-F	TCCCCCGGGATGGCAGCTATGGATTTTTGG
BD-SlDREB3-R	AACTGCAGTTATAGAGAGGCCCAATCAATTTC
pAbAi-*SlCYP707A2*-1-F	GGGGTACCGTTGTTATTTTTATGAATAGTGATTATTGTC
pAbAi-*SlCYP707A2*-1-R	GCGTCGACGTTTGGTACGAAGTAAAATATTTTCTG
pAbAi-*SlCYP707A2*-2-F	GGGGTACCGTTGTTAGCTCATGCTTATAAGATTG
pAbAi-*SlCYP707A2*-2-R	GCGTCGACCATCATGAATAAAGTGTTTCTTAACTG
pAbAi-*SlCYP707A2*-3-F	GGGGTACCTAAATCAACGGATAAGATCGTGT
pAbAi-*SlCYP707A2*-3-R	GCGTCGACTGAGATATTGGTATCTTTATCGTCTAA
pAbAi-F	TTTTTGTTCTGTGCAGTTGGG
pAbAi-R	CATGGCAGTTTGGAGGTCTCT
SlMYB78-like-nGFP-F	GCTCTAGAATGGATTATCATGGTCAGAAAATT
SlMYB78-like-nGFP-R	CGCGGATCCCATGTCATTGTTCAATTGTTGTAAG
SlDREB3-cGFP-F	TGCTCTAGAATGGCAGCTATGGATTTTTGG
SlDREB3-cGFP-R	CGGGGTACCTAGAGAGGCCCAATCAATTTCA

Table S2. Promoter analysis of *SlMYB78-like* gene.

cis-acting	Sequence	Fucntion	Number
G-box	TACGTG	Light response	4
CAACTG-motif	CAACTG	Drought response	2
ABRE	ACGTG	Abscisic acid response	2
NGNCA-motif	NGNCA	Methyl jasmonate response	1
DOFCOREZM	AAAG	Pollen-specific activation	4
LELAT52	AGAAA	Pollen-specific activation	2

Table S3. Analysis of physical and chemical properties of SlMYB78-like protein.

SlMYB78-like	
Gene ID	Solyc05g053330
Molecular weight (kDa)	36.923
Number of amino acids (aa)	318
Molecular formula	$C_{1605}H_{2478}N_{476}O_{502}S_{14}$
NCBI access number	XP_004239882
Isoelectric point	6.31
Instability coefficient	54.95
Average hydrophilicity	−0.893
CDS Length (bp)	957
Subcellular localization prediction	Nucleus
